# SDN-Based Survivability Analysis for V2I Communications

**DOI:** 10.3390/s20174678

**Published:** 2020-08-19

**Authors:** Li Jin, Guoan Zhang, Hao Zhu, Wei Duan

**Affiliations:** School of Information Science and Technology, Nantong University, Nantong 226019, China; jin.li@ntu.edu.cn (L.J.); searain@ntu.edu.cn (H.Z.); sinder@ntu.edu.cn (W.D.)

**Keywords:** multi-path transmission control protocol, probability model checker, software-defined network, survivability, vehicle-to-infrastructure (V2I)

## Abstract

In vehicle-to-infrastructure (V2I) communications, various failures in the dynamic movement pose serious link interruptions. To study the continuous service quality for the V2I network when this issue happens, this paper proposes a survivability analysis and establishes the communication architecture for software-defined network (SDN)-based V2I communications. With the controllable advantages of SDN centralized management, the multi-path transmission control protocol is used to seamlessly switch the transmission information between the V2I links of each vehicle node. Specifically, according to the analysis of specific fault types for V2I links, the definitions of SDN-based V2I survivability is provided to establish the corresponding survivability mode. To further verify the survivability model, a full-state search is adopted by means of probability model checker PRISM. In addition, multi-directional probability and expected reward evaluation analyses are carried out from the point of view of time. The simulation results show that, with the failure of multiple V2I links, the network quality of service (QoS) correspondingly declines, but the network still survives, due to the multi-path transmission control protocol (MPTCP) action. Moreover, with a high fault repair rate, the service performance and survivability of the network is improved rapidly.

## 1. Introduction

Survivability refers to the ability of the system to recover in time after the occurrence of abnormal events. It is a promising method to solve the problems of faults and various attacks in vehicle communication networks. The goal of survivability research is to analyze and evaluate the survival ability of vehicle networks, in order to find out the hidden dangers and weak links in the network and take a series of effective measures to improve the survival ability of network links.

The effective communication between the vehicle and roadside infrastructure (vehicle to infrastructure, V2I) in vehicle networks is an important factor for the road traffic convenience and safety, where the roadside infrastructure mainly includes a roadside unit (RSU), wireless access point (Wi-Fi), base station (BS, for WIMAX/LTE/4G/5G/6G), and so on. Via the roadside infrastructure, the vehicle personnel can search the optimal path to the destination, understand the real-time traffic information, transmit the current emergency information, download entertainment and news video information timely, and so forth. Thus, the connectivity of each communication link in V2I networks is key to ensure the normal operation of the whole vehicle network, and it is also reflected in the survivability of the vehicle network. It can be observed that, in the actual process of V2I network communication, due to the high-speed mobility of the vehicle, there is various randomness and uncertainty when connecting to infrastructures, meanwhile some abnormal conditions are usually encountered, leading to an interruption or failure of the link connection. These factors include encountering a denial of service (DoS) attack [1], channel fading, high-level objects, human accidents, natural disasters, and so on. In addition, the frequent switching between V2I links also provides the problem of communication delay or failure. Aiming to solve these problems, it is necessary to analyze the survivability for V2I communications to achieve the maximum transmission performance.

On the other hand, software-defined network (SDN)-based vehicle networking refers to the programmable design and flexible management under the control of the software program center [2,3]. This mobile management mechanism can be effectively adapted to the dynamic change of vehicle networks, which is more suitable for future vehicle networks based on 5G and 6G technologies than the traditional fixed management mechanism. Due to the advantage of a centralized control for SDN, the link connectivity can be effectively controlled in vehicle networks. More specifically, a SDN central controller can effectively switch to other idle links to continue the data communication, when the link connected to the vehicle has a fault problem and is waiting to be repaired. In these link-switching processes, switching mechanisms under the same or different access technologies can be used to complete the switching. It is known that the previous switching mechanism uses a transmission control protocol (TCP) and a one-to-one internet protocol (IP) path to switch frequently via a single network interface, resulting in a possible seamless connectivity along with the latency. Recently, a new switching mechanism was investigated by using multi-path transmission control protocol (MPTCP), with advantages that include making no changes to the original roadside infrastructure but simply configuring the vehicle with multiple network interfaces and IP paths, it can satisfy the one-to-many link seamless switching and significantly improve the network performance.

In recent years, many researches related to MPTCP have been applied to emerging networks, such as vehicle networks, future long term evolution (LTE) networks, an 5G networks [4,5,6,7,8]. In Reference [4], the authors described how MPTCP can effectively utilize the current radio technology and switch robustly to keep the vehicle connection and improve the network performance. Due to that, MPTCP is one of the most important candidate protocols to improve end-to-end (E2E) communication reliability, elasticity, and bandwidth efficiency in 5G mobile networks. The authors in Reference [5] introduced the applications of MPTCP to LTE networks in detail. In Reference [6], the MPTCP congestion control extension algorithm for LTE networks is proposed with the goal of aggregating the available bandwidth of multiple paths, as well as that it can avoid the common single-path TCP transmission of shared paths attack behavior. In addition, the combination of MPTCP and SDN in V2I networks within the vehicle short-distance communication is proposed in Reference [7], where the connectivity between the vehicle and Wi-Fi, vehicle and RSU are simulated by using Mininet-WiFi. Moreover, the connectivity performance is also measured from three indexes: packet loss rate, round-trip time, and average throughput, respectively. In Reference [8], the authors proposed the use of MPTCP to communicate reliably on the Internet of Vehicles (IoV).

It is worth noting that, in this paper, the survivability refers to the service quality of the system to complete the key tasks in time when the software and hardware fail, which not only pays attention to the overall security of the network system but also ensures the timely and effective transmission of the key information. On the basis of this, the study of survivability has been applied in various networks, such as information systems [9], wavelength division multiplexing (WDM) fiber optic networks [10,11], asynchronous transfer mode (ATM) networks [12], internet [13], military communications networks [14], satellite communications networks [15], wireless sensor networks [16,17,18], and vehicular ad-hoc networks (VANETs) [19]. Moreover, the research on survivability mainly includes the definition of survivability, the model of survivability, and the method of survivability analysis. The definition of survivability varies according to the application environment [20,21,22,23,24,25]. The present survivability models are mainly the Markov model, Markov reward model, semi-Markov model, and stochastic graph model [21,24,25,26,27,28,29,30]. To further improve the communication link survivability when the network fails, some effective modeling methods are introduced in the literature [31,32,33,34,35]. In addition, survivability analysis mainly includes quantitative analysis, in which quantitative analysis is presented in a numerical form and the performance is more intuitive. 

Most of the present researches on the improvement of the network performance method are mainly to evaluate the network operations from the point of view of a theoretical algorithm and simulation. To the best of our acknowledge, few studies focus on the research and applications of SDN- and MPTCP-based vehicle networks, especially for V2I network communications. Moreover, as one of the evaluation indexes of the network performance, survivability is less applied in the vehicle network, which refers to the ability of the network to still transmit tasks (or continuous service) after the failure and has a certain research value in certain kinds of vehicle networks. Therefore, it is necessary to study vehicle network survivability in the process of a random dynamic change of vehicle network topology and the failure of vehicle network links caused by various uncertain factors. With the above observations, our motivation is to analyze and evaluate the survivability in different vehicle network communication models and propose effective survivability models to provide the vehicle network survivability in abnormal situations. This paper proposes to use the probabilistic checking method to accurately analyze and evaluate the survivability of a SDN-based vehicle network from the point of view of network link failure (including software and hardware failure) and recovery. The contributions of this work can be summarized as follows:

Because the research on the survivability focuses on the direct communication between vehicle to vehicle (V2V) and V2I networks, a MPTCP mechanism for SDN-based vehicle network survivability model is proposed. In the action of the SDN central control and MPTCP, the link between each V2I can be switched seamlessly, leading to that results in the vehicle network still have some survivability under abnormal conditions (i.e., software or hardware is destroyed), and the survivability of the network is improved rapidly for a timely repairing mechanism.

Current survivability research methods normally adopt the mathematical theory derivation and simulation, which consume a lot of time and energy. Therefore, in this work, we propose time-based attributes by using the probability model detection. During analyses, by using probabilistic model detector PRISM [36,37,38], we can not only automatically search all the state space but also use continuous stochastic logic (CSL) to define the properties of the proposed model at various angles, verify the model, and obtain accurate numerical analysis results. The proposed method avoids the complicated mathematical derivation process and carries out the automatic mathematical accurate calculation to the complex survivability model. In the meanwhile, it has a simple and high efficiency characteristic and a certain reference value to the other network survivability research.

SDN-based vehicle networks currently focus more on reliability and security research but not for survivability. This paper proposes a survivability study for a SDN-based vehicle network. Since that survivability pays attention to the overall security performance of the network system, considering whether the system can "survive" under the accident of attack and hardware and software failure. Therefore, this paper comprehensively considers the software and hardware failure problems that may be encountered in the whole V2I network, injects emerging MPTCP protocol into the survivability comprehensive model, and analyzes the survival performance of the proposed model.

Due the use of single form Markov chain modeling for current survivability models, this paper considers two fault types that exist in a SDN-based vehicle network that lead to the failures of V2I link communications, these are, the vehicle node fault and the link fault. In what follows, the survivability model of these two fault types is established separately and the survivability models are considered. From the proposed survivability comprehensive model of the network, the analysis results show that the model satisfies the survivability property definitions.

The remainder of this paper is organized as follows. Section 2 describes the proposed SDN-based V2I vehicle network. Section 3 proposes the survivability definition of the V2I vehicle network. Section 4 provides a probabilistic model checking approach to analyze the survivability of the V2I network. Section 5 concludes this paper and provides possible future works.

## 2. SDN-Based V2I Network Communications

**Figure 1:**
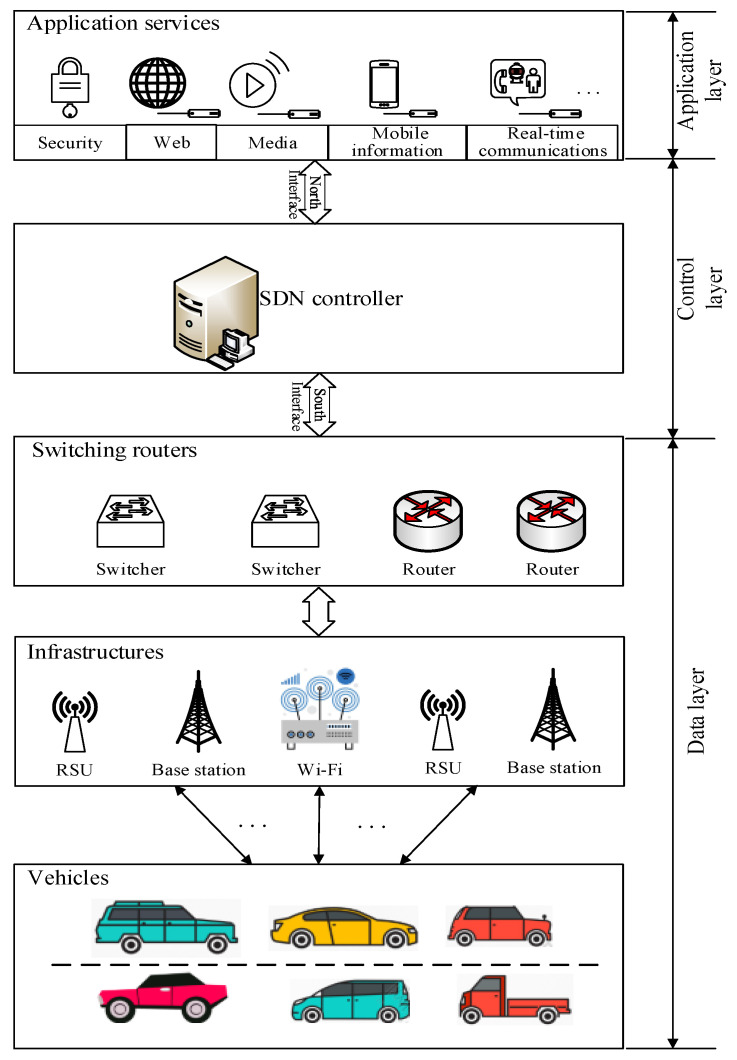
SDN-based vehicle network architecture.

The SDN-based vehicle network architecture is shown in Figure 1, which is divided into three layers: the data layer, control layer, and application layer. For the data layer, there are three elements: the vehicle, infrastructure, and switching route. The switching between communication links in V2I networks is completed through switches and/or routers. For the control layer, the SDN controller serves as a network center management part, separating the network control and the network topology of the vehicle networks, where the original network architecture extricates from the hardware control, instead of a software programmable design to centralize the dynamic management of various network parts. By using the Open Flow protocol at the south interface, it can be connected to various switching or routing devices in the data layer. Particularly, its north interface connects each application service and coordinates and controls each application service program to carry on effectively. On the other hand, for the application service layer, it mainly includes applications of the vehicle network: the security application service of the vehicle network, web service, media service of watching or downloading video, mobile information service of querying the electronic map in real time, real-time communication service, and so on. Each of these applications is controlled by the SDN controller.

**Figure 2:**
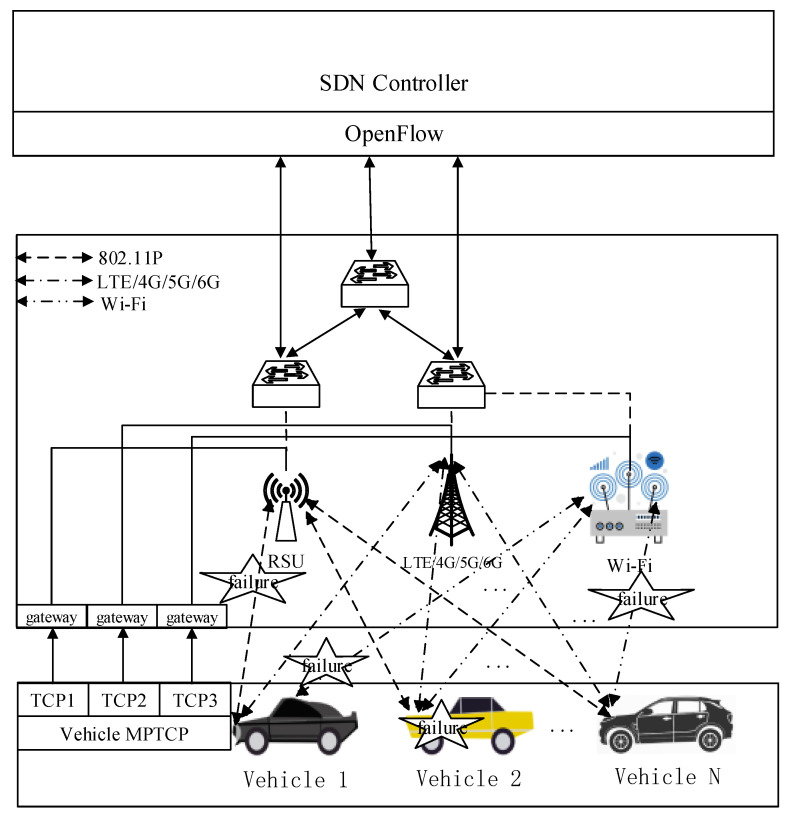
SDN-based V2I network communication model.

To analyze and study the survivability of SDN-based V2I vehicle networks, we establish a V2I network communication model based on the vehicle network architecture, which is shown in Figure 2. In this V2I network communication model, each vehicle equips three V2I communication links in three directions, namely the vehicle-to-RSU connection, vehicle-to-Wi-Fi connection, and vehicle-to-BS (such as LTE/4G/5G/6G) connection. In addition, the connection between the vehicle and RSU is carried out through the IEEE 802.11P protocol, which guarantees short-range communication between vehicle and vehicle. Moreover, vehicles and Wi-Fi IEEE802.11 standards are adopted to connect the vehicle network interface via the wireless access point (AP). For the MPTCP application, to improve the quality of network services, it is assumed that each vehicle involves multiple network interfaces. Each of them switches through MPTCP ports. The transfer between the TCP1, TCP2, and TCP3 interfaces in the MPTCP is controlled and switched by the Open Flow protocol in SDN central controller. In the Figure 2, due to the high speed movement of vehicles, there are some uncertain abnormal factors, such as the malicious vehicle intrusion or denial of service attack, channel fading, man-made accidents, natural disasters, and so on. All of these events can possibly lead to a breakdown for the V2I network and communication link. When one of the V2I links fails, MPTCP immediately responds by switching to one of the other two idle links to continue communication until the fault link is repaired in time. If either of the other two links fail, multiple repairs should be performed until the data is restored for transmission. For our proposed analysis and study of the survivability of V2I networks, we consider the fault state, fault type, and ability of the network to continue to complete the transmission task when the failure is repaired in time. The fault state includes three V2I link faults (vehicle and RSU, vehicle and base station, and vehicle and Wi-Fi) and the failure of the vehicle node. We consider that the fault types include link failures and node failures. In addition, in order to further study the ability of data transmission after the network failure, we also define the V2I network survivability according to the vehicle network communication model and then establish the survivability mode of the V2I network.

## 3. V2I Network Survivability Definition

According to the above analysis of the SDN-based V2I vehicle network communication models, the survivability of our proposed scheme can be defined as:

Definition: The SDN-based V2I vehicle network survivability model (*VSNSM*) contains a quaternion, i.e., VSNSM = {*E*, *R*, *P*, *F*}, where the definition details of each element can be summarized as follows:

*E* is the vehicle network communication application environment, the specific network communication structure is shown in Figure 2, which is based on SDN vehicle networks. The vehicle serves as a network node and communicates with each infrastructure. The inter-link switching between them controls the MPTCP through the SDN central control section.

*R* is the specific parameter setting in the vehicle network. There are two types of faults in the vehicle network, which are defined as internal and external failures. For these two faults, the internal fault refers to the failure of the vehicle node itself, and the external fault refers to the V2I link fault. We set the V2I link failure rate as λ_1_ and the vehicle node failure rate as λ_2_. Their corresponding repair rates are represented by *µ*_1_ and *µ*_2_, respectively. It is further assumed that the number of vehicles in the vehicle network is *N*, and the number of possible failures is *n* (*n* ≤ *N*). Since each vehicle has three corresponding V2I links, the maximum number of possible link failures is set to be 3*N*. The details of specific parameters are concluded in Table 1.

*P* denotes the series of probability distributions of parameters set in the *R* under certain attribute conditions, i.e., the time boundary conditions.

*F* represents the survivability model as finite state machines of a continuous time Markov chain (CTMC), which is a quaternion as *M* = {*S*, *S*_0_, *L*, *T*}. The parameters are described as follows:

- *S* = {*S*_0_, *S*_1_, *S*_2_, …, *S_n_*, …, *S_N_*} or *S* = {*S*_0_, *S*_1_, *S*_2_, …, *S_n_*, …, *S*_3*N*_}, where *N* is a finite positive integer. Two different state sets correspond to the vehicle node fault and V2I link fault, respectively. The state transition is related to the parameters in *R*;
- *S*_0_ stands for the initial state of a finite state machine;
- L: *S* → 2*^AP^* represents the label function of atomic propositions (*AP*) which are true in *S*;
- T: $S\times S\to {ℜ}_{\geq 0}$ denotes state transfer matrix.

## 4. V2I Network Survivability Model

**Figure 3:**
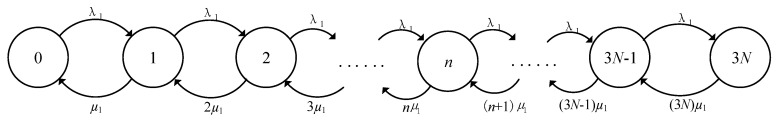
The survivability model of the V2I link failure.

According to the V2I definition of vehicle network survivability in Section 3, we now establish the survivability model of the vehicle under the V2I link failure with the specific design shown in Figure 3. To simplify the state diagram, we set “*S*_0_, *S*_1_, *S*_2_...*S_n_*...*S*_3*N*_” states in the diagram to represent the state “0, 1, 2, 3...*n*...3*N*” in the definition of survivability, respectively. The model is a CTMC with *N* vehicles on the road and their failure probability.

When any one of the V2I links fails, through the SDN control center and the MPTCP protocol, these three links of the vehicle node can be seamlessly switched to continue transmitting data. Moreover, we further define that there are 3*N* + 1 states in the survivability model of the whole vehicle network, in which the state “0” means no V2I link fault, “1” means that a V2I link fault occurs, and the subsequent digital state analogizes the corresponding V2I link fault number in turn, i.e., the maximum number of link failures in the whole network is 3*N*. At the same time, with the occurrence of a V2I link failure, in order to ensure the continuous operation of the V2I network, the network will respond to the corresponding timely repair function. According to the survival definition, the repair rate of a V2I link failure is set as *µ*_1_. The repair rate after two V2I link failures is 2*µ*_1_. Similarly, the repair rate after n V2I link failure is *nµ*_1_, and the repair rate after 3*N* V2I link failures is 3*Nµ*_1_. In this model, the number of failures corresponding to different states is repaired to different degrees.

The transient probability of the survivability model is the probability at any t time. It reflects the short-term survivability of vehicle networks. It is expressed as *P_i_*(*t*). When the initial time *t* = 0, we have the probability *P_i_*(*t*) = *P_i_*. According to the state transition, the following transient probability equations can be obtained from:

(1) $$\frac{dP_{0}(t)}{dt}=\mu_{1}P_{1}(t)-\lambda_{1}P_{0}(t),$$

(2) $$\frac{dP_{n}(t)}{dt}=\lambda_{1}P_{n-1}(t)+(n+1)\mu_{1}P_{n+1}(t)-(\lambda_{1}+n\mu_{1})P_{n}(t),\;for\;n=1,\;2,\;\ldots ,\;3N-1,$$

(3) $$\frac{dP_{3N}(t)}{dt}=\lambda_{1}P_{3N-1}(t)-3N\mu_{1}P_{3N}(t),$$

where *t*→∞, and they will tend to steady state. However, the steady state probability *P_n_* = *P_n_*(*t*) is an important measure reflecting the quality of the network operation. Let the steady state probability be *P_i_* in the *i*-th state, it can be obtained from:

(4) $$\lambda_{1}P_{0}=\mu_{1}P_{1},$$

(5) $$(\lambda_{1}+n\mu_{1})P_{n}=\lambda_{1}P_{n-1}+(n+1)\mu_{1}P_{n+1},\;for\;n=1,2,\ldots ,3N-1,$$

(6) $$\lambda_{1}P_{3N-1}=3N\mu_{1}P_{3N}$$

where $\sum_{n=0}^{3N}P_{n}=1$, by calculating the above Equations (4)–(6), the steady state probability in the *i* state can be obtained as follows:

(7) $$P_{i}=\frac{\rho^{i}}{i!\sum_{n=0}^{3N}\rho^{n}\frac{1}{n!}},$$

where $\rho =\frac{\lambda_{1}}{\mu_{1}}$, if the expected number of failures in the time range from 0 to *t* is calculated as follows:

(8) $$R_{failure\_number}(t)=\int_{0}^{t}\lambda_{1}P_{i}(x)dx.$$

**Figure 4:**
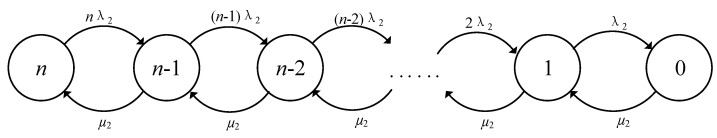
The V2I link survivability model under vehicle node failure.

The above discussions are related to the survivability model of direct failure of the V2I link, however, in the actual process, the internal fault cause of the vehicle node itself may also indirectly lead to the V2I link failure. For this situation, we establish a V2I survivability model based on vehicle node failure, as shown in Figure 4. It involves *n* states, where *n* indicates that the vehicle has no failure number, and the total number of failures at the vehicle nodes is not greater than the total number of vehicles. According to the survivability definition in Section 3, we set the failure rate of each vehicle node as λ_2_, meanwhile, for n vehicles, the possible failure rate is *n*λ_2_. For *n* − 1 vehicles, the possible failure rate is (*n* − 1) λ_2_, and so on. The corresponding failure rate for each state until the “0” state is λ_2_. At the same time, the repair rate of timely recovery after the vehicle failure is *μ*_2_. Let the transient probability of any *t* time of the survivability model be *P_i_* (*t*). Then, according to the state transition, the transient probability equations of each state can be expressed as:

(9) $$\frac{dP_{n}(t)}{dt}=\mu_{2}P_{n}(t)-n\lambda_{2}P_{n-1}(t),$$

(10) $$\frac{dP_{n-1}(t)}{dt}=n\lambda_{2}P_{n-1}(t)+\mu_{2}P_{n-3}(t)-(n\lambda_{2}+\mu_{2})P_{n-2}(t),\;for\;n=N-1,\;N-2,\;\ldots ,\;1,$$

(11) $$\frac{dP_{0}(t)}{dt}=\mu_{2}P_{0}(t)-2\lambda_{2}P_{1}(t),$$

Hence, when the time *t* tends to infinity, transient states tend to be stationary. The steady state probability is $P_{n}=\underset{t\to \infty }{lim}P_{n}(t)$. Let the steady state probability be *P_i_* in the *i*-th state, we have:

(12) $$n\lambda_{2}P_{n}=\mu_{2}P_{n-1},$$

(13) $$[(n-2)\lambda_{2}+\mu_{2}]P_{n-2}=(n-1)\lambda_{2}P_{n-1}+\mu_{2}P_{n-3},for\;n=N-1,N-2,\ldots 1,$$

(14) $$\lambda_{2}P_{1}=\mu_{2}P_{0},$$

where $\sum_{n=0}^{N}P_{n}=1$. After calculating the above equations, the steady state probability of the survival model *i* state *P_i_* can be obtained from:

(15) $$P_{i}=\frac{(\gamma {)}^{i}}{i!\sum_{n=0}^{N}(\gamma {)}^{n}\frac{1}{n!}},and\;\gamma =\frac{\mu_{2}}{\lambda_{2}},$$

If there is *k* number of faults in the V2I network, for a period of time between 0 and *t*, the expected survivability rate can be calculated as follows:

(16) $$P_{Sur}(k,t)=\sum_{i=k}^{n}\int_{0}^{t}P_{i}(x)dx,n\leq N$$

**Figure 5:**
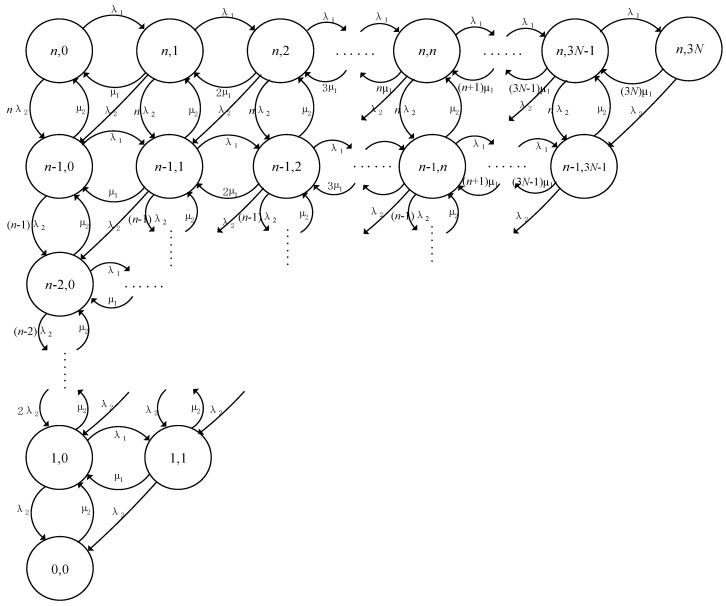
The survivability model of SDN-based V2I networks.

For practical applications, V2I vehicle networks sometimes have both internal and external faults, that is, a link failure between the vehicle and the infrastructure and a failure of the vehicle node itself. In view of this problem, we can combine these two survivability models to establish a more perfect comprehensive survivability model, as shown in Figure 5. In this model, we take into account the survivability of the above two fault situations when they occur alone but also the survivability for the case in which these two faults occur simultaneously. In Figure 5, the set state is represented by (*n*, *j*), and the parameter n indicates a failure-free number of vehicle nodes. At the same time, the parameter *n* is less than or equal to the maximum number *N* of vehicles. The parameter *j* represents the number of link failures for *j* ≤ 3*N*. An initial state is represented by (*n*, 0). The digital characters “*n*” and “0” in this state indicate that the vehicle node and V2I link are in normal condition (no failure), respectively. The state (*n*, *j*) to state (*n*, *j* + 1) indicates that the link fails, and the failure rate is λ_1_, correspondingly, the link repair rate is *μ*_1_ times the number of fault links; the state (*n*, *j*) to state (*n* − 1, *j*) indicates that there is a failure of a vehicle node, the failure rate is λ_2_ times the number of normal vehicles before the failure occurs, and its repair rate is set to *μ*_2_; the state (*n*, *j*) to state (*n* − 1, *j* − 1) indicates that the vehicle node fault and link fault occur simultaneously. The original link fault is repaired from the *j* fault number to *j* − 1 fault number. The normal number of vehicle nodes changes from the *n* original number to *n* − 1 vehicle failure nodes. In this process, one link is recovered and one of the vehicles fails. The failure rate is set to the node failure rate λ_2_, which is recovered by the repair rate *μ*_2_. According to the state transition relation of the model, we get the steady state and transient probability of the survivability of the model. Assuming that the steady state probability of the model is *P_su_*, then the probability has the following relation with the probability of each state:

(17) $$P_{su}=\sum_{i=0,j=0}^{n,3N}P_{i,j},$$

for the initial state (*n* − 1, *j*), the transient probability for *t* = 0 is *P_n_*_−1, *j*_(0) = *P_n_*_−1,*j*_. Hence, we have:

(18) $$P_{n-1,j}=\frac{3N-j}{n}P_{n,j}+\frac{j+1}{n}P_{n,j+1},$$

where j=0, 1, 2,…n…3N−1, let the transient probability of the model in [0, *t*] time be *P_su_* (*t*), then we can get:

(19) $$P_{su}(t)=P_{n-1,3N-1}(t)+P_{n,3N}(t).$$

On the basis of the above survivability model, the following sections will focus on the probability model checking and validation of the survivability with PRISM. Moreover, according to the state transition and arrival situation, the relevant survival attribute formula is defined by CSL, and the survivability probability analysis and comparison are provided under various time boundary conditions.

## 5. Verification and Analysis of V2I Networks Survivability Model

### 5.1. V2I Networks Survivability Model Verification

In this subsection, we use probabilistic model checker PRISM 4.6 for the model checking and validation. During this process, the survivability model is first designed by the PRISM programming language through the probabilistic model detection platform, and then the model is validated. During the verification process, the model checking system will automatically search all the state space and also automatically carry out state statistics, state transfer statistics, node statistics in the MTBDD, terminal node statistics, model establishment time calculation, iterative algebraic calculation in the source state reaching the target state and its iterative time calculation, and so forth. The validation of the survivability model is shown in Table 2. All the results are obtained for the given parameters as λ_1_ = 0.1, λ_2_ = 0.1, *μ*_1_ = 0.1, *μ*_2_ = 0.1, and *T* = 50, respectively. In Table 2, as the *N* of the maximum number of vehicles increases, the number of model states and state transitions will correspondingly increase. Meanwhile, the number of nodes in the Multi-Terminal Binary Decision Diagram (MTBDD) [38] and the number of terminal nodes also increase accordingly. The time of model building, number of iterations, and iteration time increase with the increased states. The model checking results verify the correctness of our proposed survivability model.

### 5.2. Analysis of Maximum Survival Probability of V2I Networks

To further analyze the survivability model, we define the relevant CSL [39,40] property formulas through the property syntax and semantic rules of the probabilistic model checker to study the survivability model from multiple angles.

In PRISM, the probability calculation in the definition CSL probability property formula is carried out by the operation P, which has the following form:

(20) P=?[pathprop],

where “*P*” represents the probabilistic results, the symbol “?” denotes the probability calculation, and “*pathprop*” indicates the path property. By this way, the maximum survival probability of the V2I network is analyzed on the basis of the time boundary. The property formula is defined by CSL as follows:

(21) $$P_{sur\_\max}=?[trueU<=T(n=N)\&(j=3*N)\{(n<N)\&(j<3*N)\}\{\max\}].$$

The formula indicates the maximum survival probability of the V2I network within the time boundary *T* = 20, when the state (*n*, *j*) reaches the target state from the initial state. From Equation (20), we can analyze the survivability of the V2I network from the angle of the influence of different parameters in the survivability model. To distinguish the survival probability under each condition, the experimental simulation is represented by *P_sur_max1_*, *P_sur_max2_*, *P_sur_max3_* and *P_sur_max4_*, respectively.

**Figure 6:**
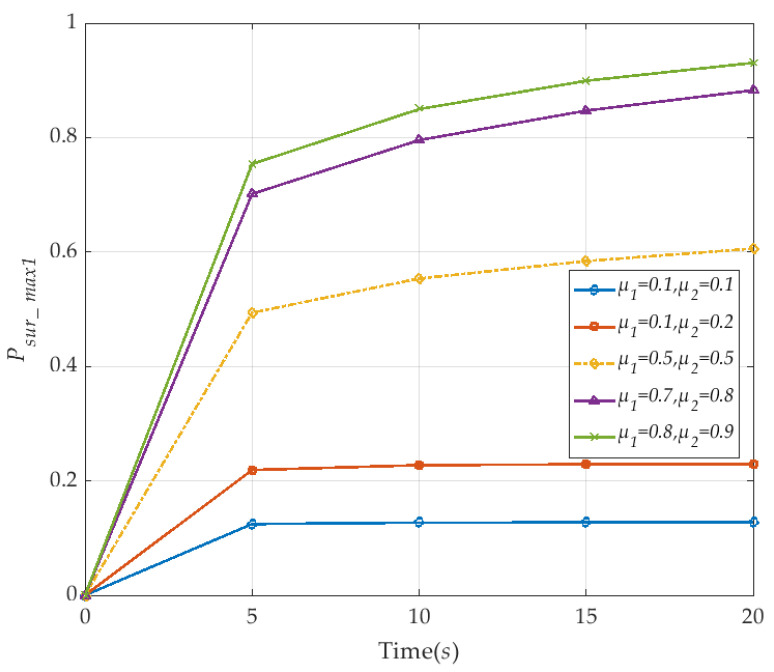
The maximum probability of V2I network survivability with respect to the time (corresponding to *μ*_1_ and *μ*_2_).

In Figure 6, we discuss the variation of the survival probability of V2I network with time. To facilitate analysis, the number *N* of vehicles in the diagram is 10, the V2I link failure rate λ_1_ and vehicle node failure rate λ_2_ are both considered as a fixed value. It is observed that the survival probability *P_sur_max1_* of the V2I network also increases as the corresponding repair rate *μ*_1_ and *μ*_2_ increase from 0.1 to 0.9, respectively. When both repair rates are small (e.g., *μ*_1_ and *μ*_2_ are 0.1, 0.2), the survival probability *P_sur_max1_* of the V2I network is low. The survival probability *P_sur_max1_* does not greatly change in 5 s, and basically remained at the stable value, and after the repair rate of both increased to 0.5, the survival probability *P_sur_max1_* with the change of time also increased rapidly. After a certain period of time, the survival probability *P_sur_max1_* of the V2I network achieves maximum value 1. The results show that the reachability of each state in the survivability model satisfies the requirements of defining properties and achieves the maximum survival probability.

**Figure 7:**
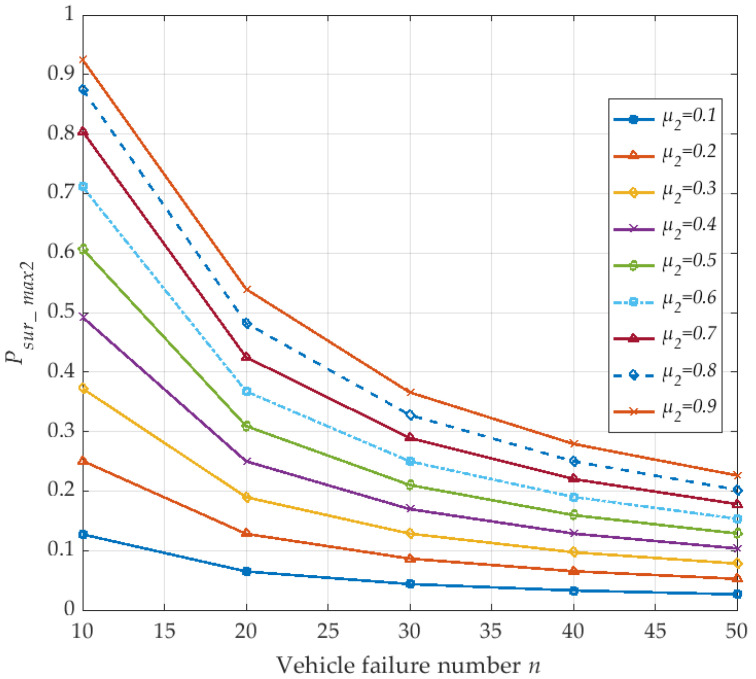
The maximum survival probability of the V2I network with vehicle failure number *n* (corresponding to *μ*_2_).

In what following, we discuss the survival probability *P_sur_max2_* of the V2I vehicle network with the number of vehicle failures. Since the number of failures that exist in different vehicle nodes is considered, the failure of the vehicle node will indirectly lead to an interruption from the corresponding V2I network communication link of vehicles. Therefore, we analyze the survival probability *P_sur_max2_* of V2I network under different vehicle failure numbers *n*, the specific situations are shown in Figure 7. We set λ_1_ = λ_2_ = 0.1, the number of vehicles is *N* = 50, and the number of vehicle failures *n* ≤ *N*. The survival probability *P_sur_max2_* of the V2I network under different vehicle node repair rate *µ*_2_ is compared in the Figure 7. The results show that the survivability probability *P_sur_max2_* of V2I network decreases with the increase of failures. In addition, with an increased vehicle node repair rate *µ*_2_ from 0.1 to 0.9, the V2I network survivability probability *P_sur_max2_* also increases. The validation results meet the property definition requirements.

**Figure 8:**
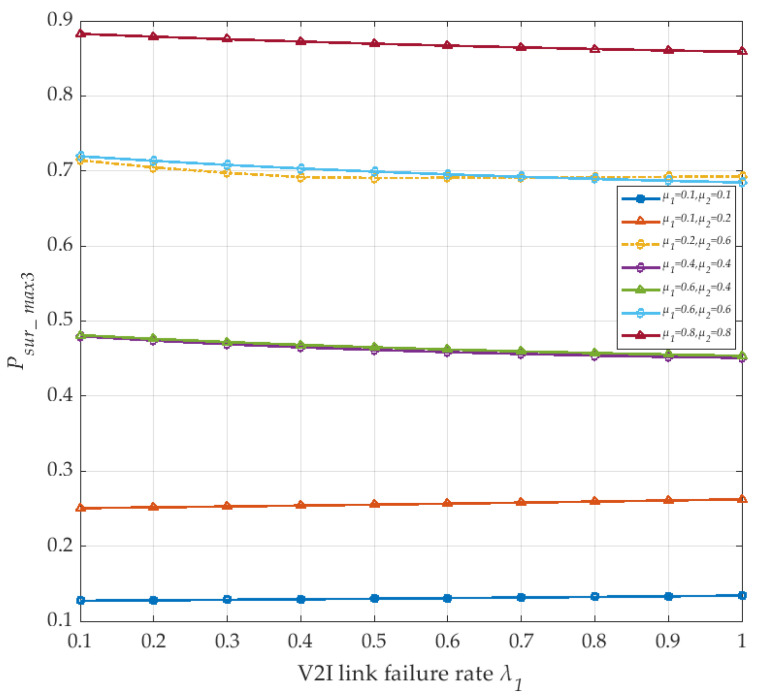
The maximum survival probability of the V2I network with link failure rate λ_1_ (corresponding to *μ*_1_ and *μ*_2_).

For the survivability model of the SDN-based V2I vehicle network, we consider the occurrence of various anomalies leading to network link failure. Thus, the V2I link failure rate λ_1_ and vehicle node failure rate λ_2_ are introduced into this model. All of these factors will directly and indirectly affect the connectivity of the vehicle network communication links. Hence, the change of λ_1_ or λ_2_ has a certain influence on the survival probability of the V2I networks. We discuss the survival probability of the V2I network in detail for different values of the parameter λ_1_, as shown in Figure 8. The number *N* of vehicles is set at 10, and λ_2_ remains 0.1. It is observed from Figure 8, with an increased repair rate *μ*_1_ and *μ*_2_, that the V2I link failure rate λ_1_ increases, especially when the repair rate of the two situations is greater than 0.4, the corresponding single curve (such as pink) shows a slight decreasing trend. The overall survival probability *P_sur_max3_* of the V2I network increases with the increased repair rate *μ*_1_ and *μ*_2_, due to the effect of MPTCP, where the repair rate *μ*_1_ dominates the repair degree of each link. Moreover, it is also shown that with an increased *μ*_1_, each V2I link still switches to each other to transfer information according to the link idle situation. Therefore, one can see that the V2I network can maintain a certain survival ability and continue to provide services for the transmission task.

**Figure 9:**
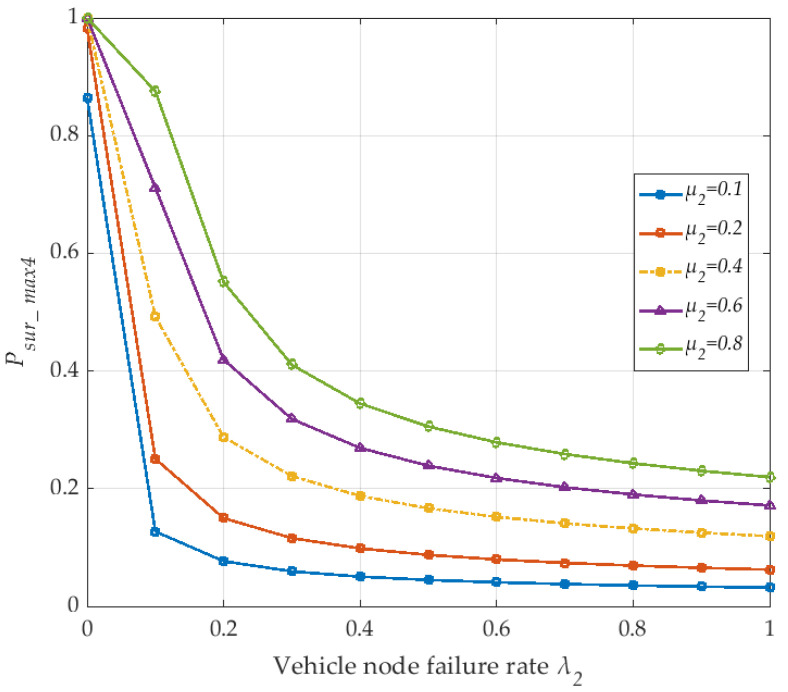
The maximum survival probability of the the V2I network with link failure rate λ_2_ (corresponding to *μ*_1_ and *μ*_2_).

Finally, due to the failure of the vehicle node affecting the survivability of the V2I network, we discuss the V2I of the network survival probability *P_sur_max4_* under the change of the failure rate λ_2_ with the vehicle node, as shown in Figure 9. Failures of vehicle nodes will lead to interruptions of V2I network communication links. The single curve shown in Figure 9 shows that the survival probability *P_sur_max4_* of the V2I network decreases with an increased failure rate λ_2_ of the vehicle node. However, the overall survival probability of the V2I network increases with the increased repair rate *μ*_2_. Moreover, in Figure 9, one can see that the entire V2I network has a certain survivability even at a minimum repair rate of 0.1. Although the survival probability *P_sur_max4_* is relatively low, the network still has a certain survivability. Therefore, the MPTCP technology for vehicle network nodes in the SDN-based V2I network has advantages in maintaining network survival performance.

### 5.3. Instantaneous Evaluation Analysis of the V2I Network Survivability

The V2I network survivability model is a CTMC, which has an expected reward at some instantaneous time. It is usually called an instantaneous expected value. The CSL property is defined as follows:

(22) R=?[I=T].

This expression represents the expected value of the V2I network at *T* instantaneous time. We first discuss the instantaneous expected value of the number of link repairs in V2I the network at different repair rates when the failure rate and number of vehicle failures are constant. The specific CSL formula is expressed as:

(23) R{“Expected repair_number”}=?[I=T].

**Figure 10:**
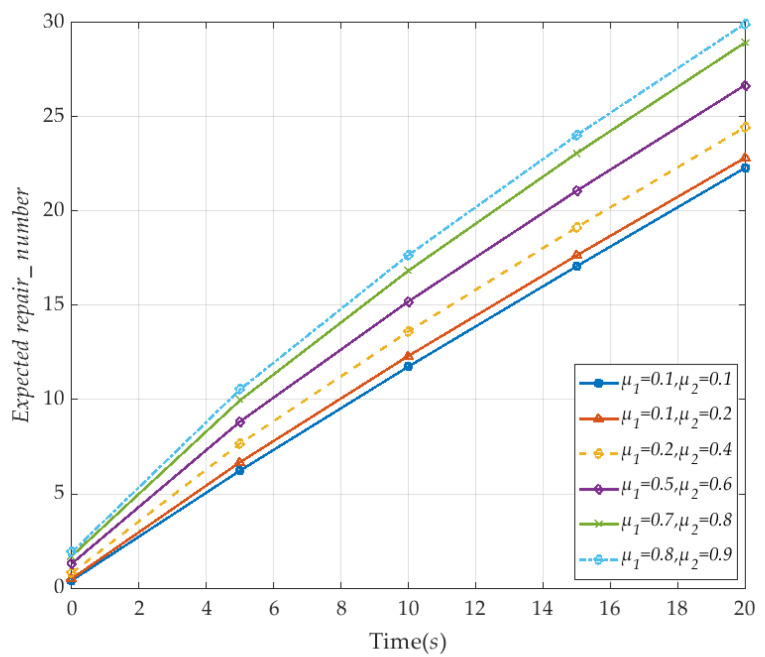
The instantaneous expected repair number of the the V2I network with time (corresponding to *μ*_1_ and *μ*_2_).

We set the number of link failures to be 10, λ_1_ and λ_2_ are set to be 0.1, the instantaneous time *T* is 20 s. Specific analytical values are shown in Figure 10. It shows that the instantaneous expected value of the V2I link repair increases with the increased repair rate *μ*_1_ and *μ*_2_. When *μ*_1_ = 0.8 and *μ*_2_ = 0.9, the expected value of repair numbers can reach the maximum value 30. The results show that each state achieves the target state to satisfy the requirements of the defined properties and the instantaneous expected reward. The V2I network can achieve maximum survivability by the instantaneous expected evaluation.

Furthermore, by means of the instantaneous property formula, we also analyze the expected number of link failures in V2I the network under different failure rates. The specific CSL formula is expressed as:

(24) R{“Expected failure_number”}=?[I=T].

**Figure 11:**
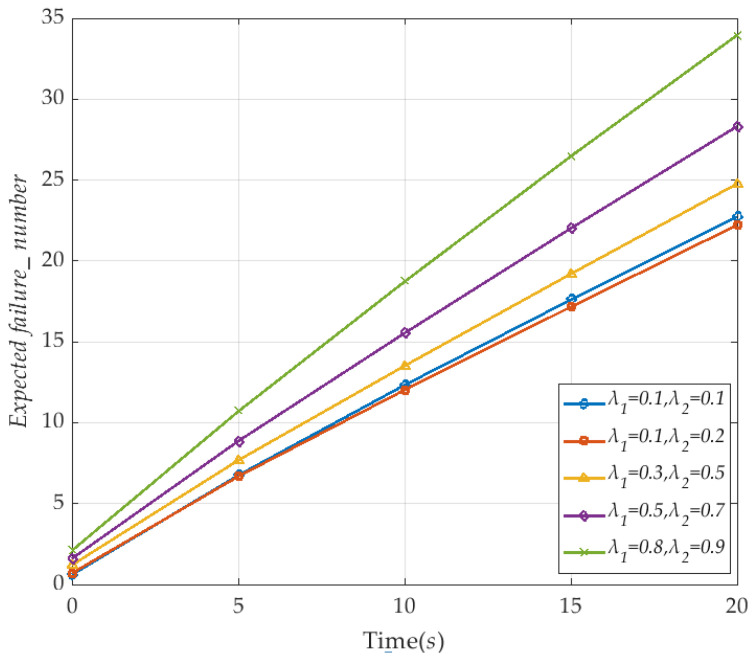
The instantaneous expected failure number of the the V2I network with time (corresponding to *μ*_1_ and *μ*_2_).

Time *T* is also set as 20 *s*, the repair rate *μ*_1_ and *μ*_2_ as 0.2, and the number of vehicles as 10. The specific values are shown in Figure 11. It shows that, as the V2I link failure rate λ_1_ and the vehicle node failure rate λ_2_ gradually increase, the number of instantaneous failures in the V2I network becomes greater, and the expected value also increases. Thus, the instantaneous expected evaluation of network survivability is reduced.

### 5.4. Instantaneous Evaluation Analysis of the V2I Network Survivability

The CTMC state model will obtain the cumulative expected reward after a period of time, which is the expected reward based on the time boundary, as defined below:

(25) R=?[C≤T],

where the formula represents the expected reward of V2I network over *T* time, and its expected value is calculated using time *T* as the boundary for cumulative rewards. Similar to the instantaneous expected value analysis, we first analyze the expected reward of the number of link repairs in the V2I network at different repair rates, when the failure rate and number of vehicle failures remain are fixed. The specific CSL formula is expressed as:

(26) R{“Expected reward of repair_num”}=?[C≤T].

**Figure 12:**
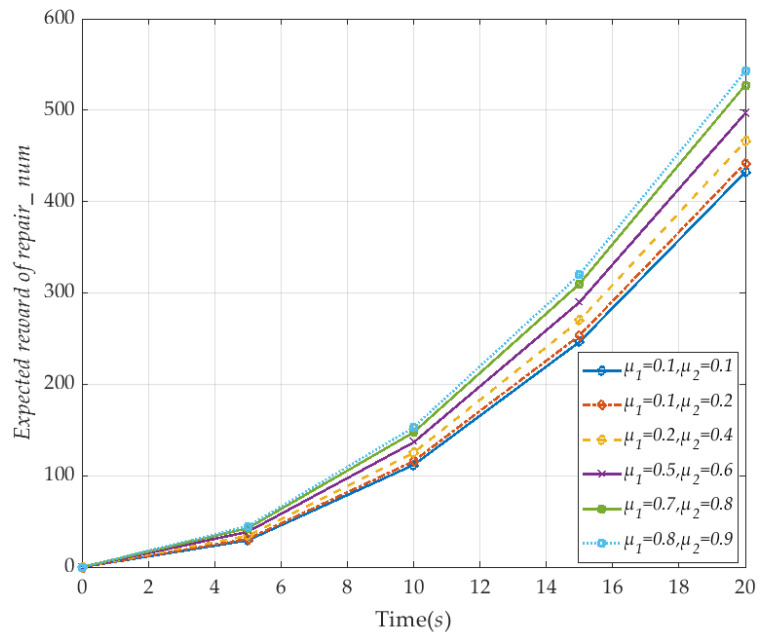
The expected repair number reward of the the V2I network with time (corresponding to *μ*_1_ and *μ*_2_).

The specific numerical results are shown in Figure 12. The number of link failures are set at *n* = 10, the λ_1_ and λ_2_ are set to be 0.1, instantaneous time *T* is 20 *s*. The figure shows that the expected link repair number of V2I network is improved with the increased repair rate *µ*_1_ and *µ*_2_ in the *T* time boundary. This indicates that the number of V2I links available in the network increases. As a result of MPTCP, each vehicle seamlessly switches between its effective links, and the V2I network service quality is improved, so that the V2I network survival ability is enhanced, and the survivability is improved. 

Finally, we discuss the expected number of link failures in the V2I network under different failure rates λ_1_ and λ_2_ within the *T* time boundary. The specific CSL formula is expressed as:

(27) R{“Expected reward of link_failure_num”}=?[C≤T].

**Figure 13:**
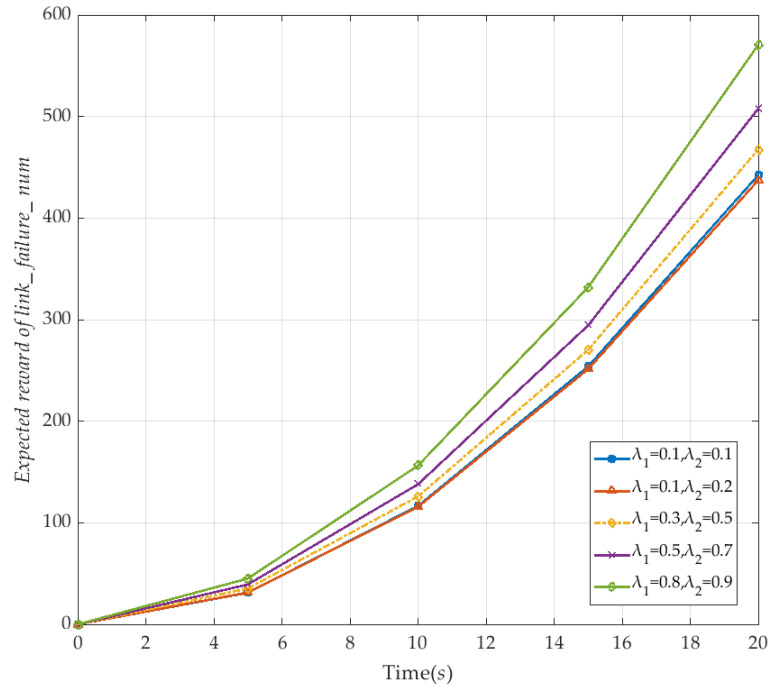
The expected reward of the link failure number in the V2I network with time (corresponding to λ_1_ and λ_2_).

In order to match the number of failures with the instantaneous expectation, the time boundary *T* is also set to 20 s, the repair rate *μ*_1_ and *μ*_2_ is set to 0.2, and the number of vehicles is set to 10. Specific values are shown in Figure 13. It is easy to see that, under the time boundary *T*, the expected reward of the failure numbers are calculated continuously on the basis of each instantaneous value until the set time boundary. The expected number of failures in V2I network increases with the increased failure rates λ_1_ and λ_2_. This indicates that the V2I network service quality is degraded, which leads to a downward trend in the expected evaluation of the network survivability of the V2I network.

## 6. Conclusions

This paper proposed a probabilistic model-checking method to quantitatively analyze the survivability for the SDN-based V2I network. We investigated the SDN-based vehicle network communication architecture and established the SDN-based V2I vehicle network communication model, in which the vehicle node fault and link fault type were considered. On the basis of the proposed communication model, we provided the survivability definition of SDN-based V2I vehicle networks. Combined with MPTCP protocol, we proposed a Markov chain-based survivability model of the SDN-based V2I network. Moreover, a probabilistic model-checking method was investigated to validate the V2I network survivability model. In particular, we analyzed the probability and expected reward under various conditions from the point of view of time. The analysis results showed that, under the action of MPTCP, multiple V2I failures occur, the QoS of the network decreases, but the network still has a certain survival ability. Moreover, for a higher fault repair rate, the service capacity of the network will be improved quickly, and the survivability will be further improved. In summary, the results showed that our proposed survivability enhancement method and analysis method of probabilistic model checking are effective for SDN-based V2I vehicle networks. Additionally, the proposed survivability model was limited to a certain application environment and did not realize a general survivability model. In our future research work, we will evaluate and compare the survivability of other vehicle networks’ models and summarize a suitable general model for the vehicle network survivability. Specifically, we will explore more effective strategies to improve the survivability of vehicle networks and study the survivability of vehicle networks for 5G and 6G combined with the artificial intelligence approach.

## Figures and Tables

**Table 1:** Parameter description.

Parameter	Detailed Description
*n*	failure number
*N*	vehicle number
λ_1_	V2I link failure rate
λ_2_	Vehicle failure rate
*µ*_1_	V2I link repair rate
*µ*_2_	vehicle repair rate

**Table 2:** Probability model test results of the survivability model.

Vehicle Number *N*	Model		MTBDD		Construction Time(s)	Reachability	
	States	Transitions	Nodes	Leaves		Iter. (s)	Time Per Iter. (s)
10	341	1451	456	31	0.017	41	0.000024
50	7701	37,251	2877	151	0.04	201	0.000104
100	30,401	149,501	5968	301	0.116	401	0.000162
200	120,801	599,001	12,434	601	0.454	801	0.000306
300	271,201	1,348,501	19,188	901	0.899	1201	0.000428
400	481,601	2,398,001	25,950	1201	1.669	1601	0.000565
500	752,001	3,747,501	34,384	1501	2.479	2001	0.000692
600	1,082,401	5,397,001	40,054	1801	3.549	2401	0.000857
700	1,472,801	7,346,501	51,616	2101	5.172	2801	0.001035
800	1,923,201	9,596,001	54,166	2401	6.608	3201	0.001260
900	2,433,601	12,145,501	66,320	2701	10.303	3601	0.001453
1000	3,004,001	14,995,001	71,650	3001	11.735	4001	0.001441
